# Wendan decoction for dyslipidemia: Protocol for a systematic review and meta-analysis

**DOI:** 10.1097/MD.0000000000014159

**Published:** 2019-01-18

**Authors:** Wenwei Feng, Xiaohan Ye, Hongxue Lv, Chijun Hou, Yingjun Chen

**Affiliations:** Department of Cardiology, Dongguan Traditional Chinese Medicine Hospital, Dongguan, Guangdong Province, China.

**Keywords:** dyslipidemia, protocol, systematic review, Wendan decoction

## Abstract

**Background:**

Dyslipidemia is one of the most popular metabolic diseases and an important risk factor for arteriosclerotic cardiovascular diseases. In China, Wendan decoction (WDD) has been widely used to treat hyperlipidemia. However, no systematic review has been found. In order to evaluate the efficacy and safety of WDD in the treatment of dyslipidemia, a meta-analysis and systematic evaluation are conducted.

**Methods:**

The randomized controlled trials (RCTs) evaluating the effectiveness and safety of WDD in the treatment of dyslipidemia will be enrolled. Data are mainly from 4 English databases (Pubmed, Embase, Cochrane Library, and Web of science) and 4 Chinese databases (Wanfang, CBM, CNKI, and VIP Database). The enrollment of RCTs is from the starting date of database establishment till December 15, 2018. Low density lipoprotein cholesterol is considered as the main outcome, while the serum concentrations of total cholesterol, triglyceride, high density lipoprotein cholesterol, apolipoprotein A, and apolipoprotein B are regarded as the secondary outcome. Safety indicators include liver enzyme, fasting blood glucose, and kidney function. The work such as selection of literature, data collection, quality evaluation of included literature, and assessment of publication bias will be conducted by 2 independent researchers. Meta-analysis will be performed by RevMan 5.0 software.

**Results:**

This study will provide high-quality evidence for the treatment of dyslipidemia with WDD in terms of effectiveness and safety.

**Conclusion:**

The results of the study will help us determine whether WDD can effectively treat hyperlipidemia.

**PROSPERO registration number:**

PROSPERO CRD 42018114957.

## Abstract 2

Supplemental Digital Content is available in the text

## 1 Introduction

Dyslipidemia is a common metabolic disease in modern society, mainly characterized by an increase in total cholesterol (TC), low-density lipoprotein cholesterol (LDL-C), triglycerides (TG), and a decrease in high-density lipoprotein cholesterol (HDL-C). Studies have shown that lipoprotein disorders are an important risk factor for arteriosclerotic cardiovascular diseases (ASCVDs) such as acute coronary syndrome, stable angina pectoris and stroke, and so on.^[1,2]^ Researches indicate that the cardiovascular events are reduced by 20% to 25% for every 1 mmol/L reduction in LDL-C.^[3,4]^ Therefore, lipid-lowering therapy is of great significance. Statins are widely used in clinical practice as lipid-lowering drugs. They work by inhibiting the activity of 3-hydroxy-3-methylglutaryl coenzyme A reductase and accelerating the decomposition of LDL. The side effects of these Pharmaceuticals include rhabdomyolysis, elevated transaminase levels, increased risks of new onset type 2 diabetes or Parkinson disease, which necessitates discontinuation of these drugs in some patients.^[5,6]^ In this situation, alternative medicine has attracted people's eyes as an option for lipid-lowering treatment. Many studies have shown that Chinese medicine, acupuncture and other methods have achieved good results in the treatment of hyperlipidemia.^[7,8]^ Among which, WDD is a prescription worthy of attention. It is made up of 6 kinds of traditional Chinese medicines (Pinellia ternata, tangerine peel, bamboo ru, medlar, ginger, and licorice). Animal experiments have shown that WDD can regulate the lipoprotein disorders by raising the activity of total lipase and lipoprotein lipase in the modal rats.^[9]^ A large number of clinical studies have also found that WDD is effective in the treatment of dyslipoproteinemia.^[10–12]^ However, no systematic studies have been found on the efficacy and safety of WDD in lipid-lowering therapy. This study uses a meta-analysis method to systematically evaluate the efficacy and safety of WDD in treating hyperlipidemia, which will provide strong evidence-based medicine support for its clinical applications.

## 2 Method

### 2.1 Inclusion criteria for study selection

#### 2.1.1 Types of studies

Randomized controlled clinical trials (RCTs) published in English, whether blinded or not, will be selected. No time is limited.

#### 2.1.2 Types of patients

Adult participants (older than 18 years of age) with dyslipidemia will be enrolled. The diagnosis of hyperlipidemia can be made if the patient’ blood lipids remain high 2 to 4 weeks later after his initial visit.^[1,2]^ No race, nationality, age, gender, and comorbidity are limited.

#### 2.1.3 Types of interventions

The control group was given no medication, placebo or lipid-lowering drugs (such as stains). And the experiment group was added WDD on the basis of treatment measures of the control group. The administration time of each group was no longer than or equal to 4 weeks.

#### 2.1.4 Types of outcome measures

##### 2.1.4.1 Primary outcomes

According to the guidelines for clinical research of new drugs in traditional Chinese medicine and the new AACE/ACE guideline, LDL-C is identified as the main outcome indicator.^[1,13]^ It is considered markedly effective, if LDL-C is decreased by ≥20%; effective, LDL-C decreased by 10% to 20%; invalid, if the decline level of LDL-C does not meet the above criteria.

##### 2.1.4.2 Secondary outcomes

According to the guidelines for clinical research of new drugs in traditional Chinese medicine^[1]^ the secondary outcome indications include serum concentrations of TC, TG, HDL-C, apolipoprotein A, and apolipoprotein BS.

##### 2.1.4.3 Safety outcomes

Safety indicators consist of liver enzyme, fasting blood glucose, and kidney function.

### 2.2 Search methods for the identification of studies

Four English databases (Pubmed, Embase, Cochrane Library, and Web of science) and 4 Chinese databases (Wanfang, China Biomedical Literature Database, China Academic Journal Full-text Database, and VIP Database) are to be searched. The search date is up to October 15, 2018. Search terms include hyperlipemia, WDD, and RCTs. The search formula for PubMed will be shown as an example in Appendix A (Supplemental Appendix A). Similar search strategies will be adopted in other databases.

#### 2.2.1 Searching other resources

Search engines such as Google Scholar and Baidu Academic will be applied to search relevant literature on the Internet. We will search clinicaltrial gov to attained relevant clinical studies. And the investigators would be contacted for relevant results, if necessary. In addition, citation searches will be performed manually in order to avoid missing important information.

### 2.3 Data collection and analysis

#### 2.3.1 Selection of studies

**Figure 1:**
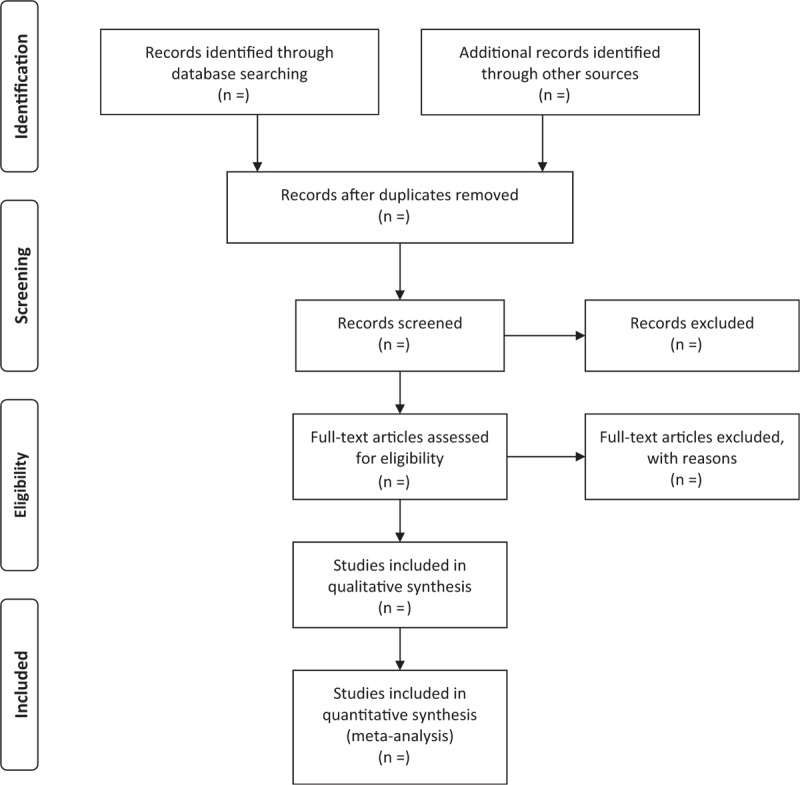
Preferred reporting items for systematic review and meta-analysis (PRISMA) flow chart.

The MedRef4.0 document management system will be used to eliminate duplicate documents by using the software's automatic check function. The screening of the literature is to be completed by 2 investigators in accordance with pre-established inclusion and exclusion criteria. The screening process includes 2 stages. The first stage is to screen the literature by reading the title and abstract. The second stage is to screen the literature by reading the full text. If the 2 investigators have different opinions when reading the title and abstract of the article, then the literature should be included for full-text reading screening. The process of literature selection and meta-analysis is presented in an adapted preferred reporting items for systematic review and meta-analysis (PRISMAP) flow diagram (Fig. 1).

#### 2.3.2 Data extraction and management

Two researchers will collect data based on a pre-established data extraction protocol. A researcher with statistical analysis experience will be invited to supervise and guide the data collection. The content of the collection includes author, publication time, research methods, interventions, sample size, measurement indicators, results, and adverse reactions. If 2 researchers have different opinions, they should discuss with each other or negotiate with another researcher. If the information in the literature is incomplete, one should contact the author for it. If the relevant data cannot be obtained, the study should be excluded.

#### 2.3.3 Assessment of risk of bias in included studies

The quality of the included literature will be evaluated according to the Jadad score.^[14]^ The evaluation includes the generation of random sequences, randomization, the implementation of blind methods by subjects and researchers, and the withdrawal and withdrawal criteria. The first 3 criteria are scored by 3 levels: appropriate, unclear, and inappropriate. The last criterion is graded on whether withdrawal and exit reasons are described. A database for evaluation is created by using Microsoft Excel. And quality scores of the literature are entered and analyzed in the database.

#### 2.3.4 Measures of treatment effect

The standardized mean difference is used to analyze the measurement data, while the relative risk is used to analyze the count data.Ninety-five percent confidence intervals will be calculated for all analyses. *P* < .05 is considered statistically significant.

#### 2.3.5 Dealing with missing data

If the information of the article is missing, we will contact the author for further information. If the necessary information is not obtained, we will use the available data for data synthesis.

#### 2.3.6 Assessment of heterogeneity

Heterogeneity is determined by heterogeneity test and expressed by I2 value. When *I*^2^ < 25%, the heterogeneity is considered small. When 25% < *I*^2^ < 50%, it means that there is moderate heterogeneity. When *I*^2^ ≥ 50%, the heterogeneity is large.

#### 2.3.7 Assessment of reporting bias

The funnel plot and the Begg test will be combined to analyze the impact of publication bias.^[15]^

#### 2.3.8 Data synthesis

Meta-analysis will be performed by RevMan 5.0 software (Version 5.3, Copenhagen: The Nordic Cochrane Center, 2014) provided by the Cochrane Collaboration. When there is statistical homogeneity between each study (*I*^2^ < 50%), the fixed effect model is used. When the heterogeneity is significant (*I*^2^ ≥ 50%) between the results of each study, the sub-layer analysis is performed to find the source of heterogeneity. A fixed effect model is used for meta-analysis when there is sufficient similarity between the results of the subgroups (*I*^2^ < 50%). However, a random effect model is used for meta-analysis if the heterogeneity between the results of the subgroups is significant (*I*^2^ ≥ 50%). Qualitative heterogeneity is used when heterogeneity is too large or the source of heterogeneity is unknown. Meta-regression analysis can be performed if there are many influencing factors and it is not appropriate to use the stratification method.

#### 2.3.9 Subgroup analysis

When more than 10 studies are conducted, subgroup analyses will be performed based on different interventions, participants, gender, duration of disease, dose, and so on. In this way, the source of heterogeneity can be better explored.

#### 2.3.10 Sensitivity analysis

Sensitivity analysis is based on sample size, missing data results, and methodological quality.

#### 2.3.11 Grading the quality of evidence

It is recommended to use the software of GRADE profiler 3.6 to analyze the quality level of the evidence. Accordingly, the results will be divided into 4 levels: high, medium, low, or very low.

## 3 Discussion

Dyslipidemia is a common metabolic disease in modern society. According to the survey, there are more than 200 million patients with hyperlipidemia in China.^[16]^ It is well known that dyslipidemia is an important risk factor for ASCVD, which brings a heavy burden to our society. Statins are currently the primary choice for the treatment of dyslipidemia. In clinical practice, we often met some patients who cannot be tolerant of statins’ side effects. These side effects include rhabdomyolysis, liver damage, and an increased risk of new-onset type 2 diabetes or Parkinson disease. At present, the optimal lipid management method for statin intolerance patients remains controversial.^[17]^ Studies have shown that WDD can not only reduce blood lipid levels but also improve the clinical symptoms such as dizziness and fatigue in patients with hyperlipidemia. However, there is currently no systematic review and META analysis to evaluate its therapeutic effects. Therefore, a high-quality systematic review and meta-analysis is necessary and the process is shown in the flow chart (Fig. 1). The researchers hope that the study will provide more convincing evidence to prove the advantages of WDD in the treatment of hyperlipidemia. However, there may be some potential shortcomings in this study. First, the dose and origin of Chinese herbal medicines included in the research may be different, and there is a risk of heterogeneity. Second, this study may involve a small sample of clinical trials that may lead to a high risk of bias. The list of PRISMAP is presented online.

## Author contributions

**Conceptualization:** Xiaohan Ye.

**Data curation:** Hongxue Lv.

**Investigation:** Chijun Hou.

**Software:** Yingjun Chen.

**Supervision:** Xiaohan Ye.

**Validation:** Wenwei Feng.

**Writing – original draft:** Wenwei Feng.

## Supplementary Material

Supplemental Digital Content
