# Supplementary material for: Wendan decoction for dyslipidemia: Protocol for a systematic review and meta-analysis
Source: Medicine (Baltimore). 2019 Jan 18;98(3):e14159. doi: 10.1097/MD.0000000000014159 (PMC6370125; doi:10.1097/MD.0000000000014159)
Supplement: Supplemental Digital Content [file medi-98-e14159-s001.docx]

**Appendix A.**

***Search strategy used in PubMed database***

#1 hyperlipemia OR hyperlipemias OR hyperlipidemia OR lpidemia OR hipidemias OR lipemia OR lipemias OR dyslipidemia OR dyslipoproteinemias OR Dyslipoproteinemia OR lipoprotein disorder

#2 wendan decoction OR wen dan decoction OR wendan tang OR wendan yin OR gallbladder warming decoction

#3 Randomized controlled trial OR clinical study OR Clin-ical Trial OR Controlled study OR Controlled Trial OR Random*Control* study OR random* Control* Trial

#1 AND #2 AND #3
